# Haplotypes in candidate genes related to nitric oxide pathway and vascular permeability associated with migraine and aura

**DOI:** 10.1007/s10194-012-0438-5

**Published:** 2012-03-25

**Authors:** Flavia Magazoni Gonçalves, Marcelo Rizzatti Luizon, Jose G. Speciali

**Affiliations:** 1 Department of Pharmacology, Faculty of Medical Sciences, State University of Campinas, Rua Tessália Vieira de Camargo, 126, Campinas, SP CEP: 13083-887 Brazil; 2 Department of Pharmacology, Faculty of Medicine of Ribeirao Preto, University of Sao Paulo, Ribeirão Prêto, SP Brazil; 3 Division of Neurology, Department of Neuroscience, Faculty of Medicine of Ribeirao Preto, University of Sao Paulo, Ribeirão Prêto, SP Brazil

Dear Editor,

In a recent article in *Journal of Headache and Pain*, Markus Schürks has reviewed current findings on migraine genetics identified by both candidate gene approaches and genome-wide association studies [1]. We would like to contribute to some candidate gene studies focused on endothelial function possibly involved in migraine pathophysiology, which reported associations of haplotypes with migraine with aura (MA) or with aura in migraine patients [2–4]. This common condition has gained much attention because it is now recognized as an established risk factor for cardiovascular disease and ischemic stroke [5].

We have studied three clinically relevant SNPs in the vascular endothelial growth factor (*VEGF*) promoter region; C^−2578^A (rs699947), G^−1154^A (rs1570360), and G^−634^C (rs2010963) and found that haplotype “AGC” was more frequent in MA than controls (*P* = 0.0023) [2]. We have also examined three clinically relevant endothelial nitric oxide synthase (*eNOS*) polymorphisms; T^−786^C (rs2070744), an Intron 4 VNTR and Glu298Asp (rs1799983), and two tagSNPs rs3918226 and rs743506. Our findings suggest that haplotypes “CCaGluG” and “CCbGluG” are associated with increased susceptibility to the presence of aura in patients with migraine (both *P* < 0.001) [3]. Finally, we have also examined two functionally relevant polymorphisms of inducible nitric oxide synthase (*iNOS*); C^−1026^A (rs2779249) and G2087A (rs2297518). The haplotype “AA” was more commonly found in MA than in patients without aura (*P* = 0.0027) [4].

Gene–gene interaction studies among candidate genes are further required in order to better elucidate the genetic basis of migraine, as previously pointed out elsewhere [6]. Therefore, we are currently performing interaction analysis among the polymorphisms previously reported [2–4]. However, our findings on *eNOS, iNOS* and *VEGF* haplotypes must be replicated in populations with different genetic backgrounds, which will further validate the role of these candidate genes associated with migraine and aura and may provide clinically relevant information to migraine susceptibility.
